# Open Access Multimodal fNIRS Resting State Dataset With and Without Synthetic Hemodynamic Responses

**DOI:** 10.3389/fnins.2020.579353

**Published:** 2020-09-29

**Authors:** Alexander von Lühmann, Xinge Li, Natalie Gilmore, David A. Boas, Meryem A. Yücel

**Affiliations:** ^1^Neurophotonics Center, Biomedical Engineering, Boston University, Boston, MA, United States; ^2^Department of Speech, Language and Hearing Sciences, Boston University, Boston, MA, United States

**Keywords:** multimodal, open access, synthetic HRF, resting, fNIRS

## Introduction

Functional Near-Infrared Spectroscopy (fNIRS) is an optical neuroimaging technology that has rapidly gained momentum within the last decades (Boas et al., 2014; Scholkmann et al., 2014; Yücel et al., 2017). It is a non-hazardous and non-invasive optical brain imaging technique that uses near-infrared light to measure local cortical concentration changes of oxygenated and deoxygenated hemoglobin (HbO_**2**_/HbR), which are associated with brain metabolism (Villringer and Chance, 1997; Ferrari and Quaresima, 2012). fNIRS has been considered a cost-effective and mobile alternative for functional Magnetic Resonance Imaging in conventional neuroscientific research. It is very suitable—and thus increasingly being used—for single trial analysis and Brain Computer Interface (BCI) applications (Matthews et al., 2008; Hong et al., 2018) as a single modality or along with Electroencephalography (EEG). While EEG and fNIRS signal processing is essential to increase the contrast to noise ratio (CNR) of measured brain responses, the nature of the signals and processing methods differ greatly. Hemodynamic brain responses in fNIRS are usually masked by local and systemic physiological confounding signals, for instance from superficial (scalp) blood flow, low frequency oscillations (Mayer waves), motion and breathing (Elwell et al., 1999; Yücel et al., 2016; von Lühmann et al., 2019). New and increasingly complex and powerful statistical methods are being developed that aim to remove the confounding factors in the signal, improve CNR and increase the detection/classification accuracy of hemodynamic responses. An objective way of validating the power of these novel methods and comparing them with the existing ones is to use an fNIRS dataset which has all the confounding signals but also a known hemodynamic brain response. One solution for this problem is to generate realistic fNIRS ground truth data by modeling a hemodynamic response function (HRF) on top of real resting state data (Gagnon et al., 2012; von Lühmann et al., 2019, 2020a,b). This approach can be used as a good approximation for realistic fNIRS signals with evoked responses, for which the ground truth is available. Generating such data is comparatively straight forward but requires prior knowledge in fNIRS signal characteristics as well as experience in fNIRS signal processing. Moreover, the use of short-separation fNIRS measurements and additional physiological signals, such as accelerometer or photoplethysmography (PPG), has been shown to enable methods that yield improved CNR (Yücel et al., 2015; von Lühmann et al., 2020a), but there are only few openly available multimodal fNIRS datasets (Shin et al., 2017, 2018) and even fewer multimodal datasets that include sufficient resting state periods to enable the approach described above. Thus, as a remedy, here we provide such a multimodal dataset with (and without) added synthetic HRF ground truth, short-separation fNIRS measurements, accelerometer, and other physiological measurement, for the data science community in order to facilitate the validation of novel methods. We also provide a simple code example to enable customization and modification of the HRF ground truth in the data.

## Methods

**Figure 1:**
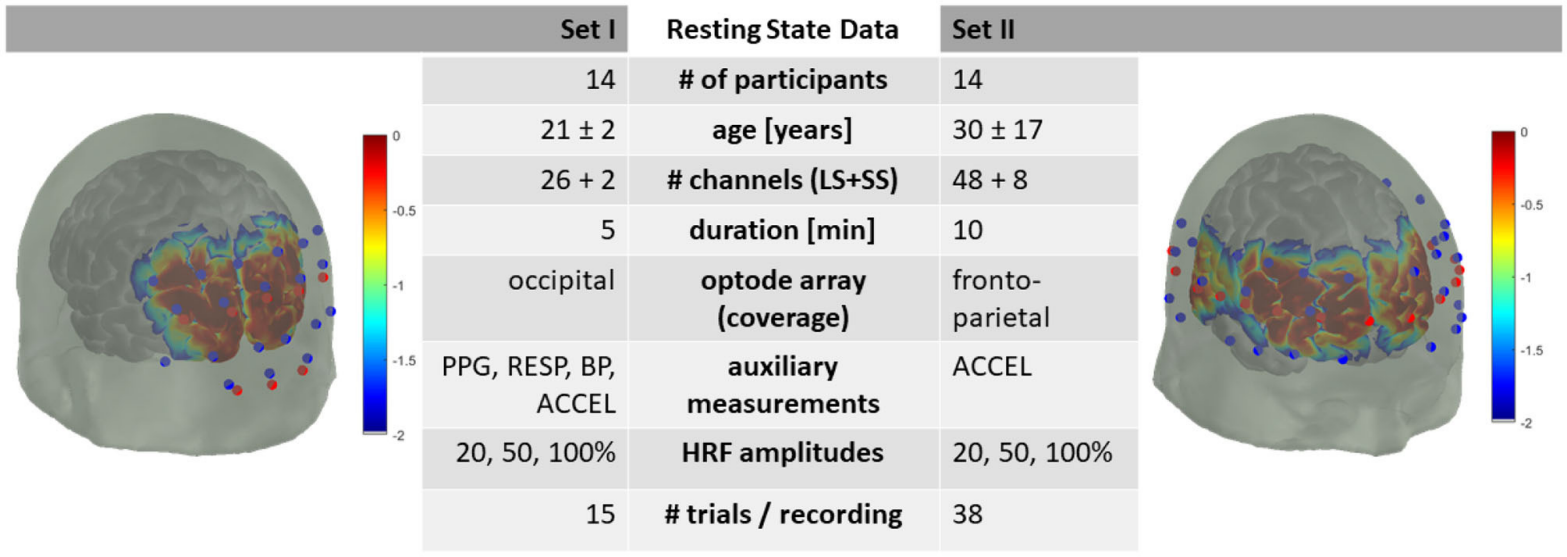
Summary of dataset metadata. Red dots are fNIRS emitters, blue dots are fNIRS detectors. LS, Long-Separation; SS, Short-Separation; PPG, Photoplethysmography; RESP, Respiration; BP, Blood Pressure; ACCEL, Accelerometer. HRF with 20, 50, and 100 % of +0.66 | −0.23 μM peak HbO_2_/HbR amplitude. Sensitivity profile in log10 dB.

The resting state data consists of two subsets: Dataset I, with 5 min resting state data from 14 participants and Dataset II, with 10 min resting state data from 14 participants. The data details follow and are summarized in Figure 1.

### Participants and Demographics

Dataset I consists of recordings from 14 healthy participants (age: 21 ± 2 years; 11 male/3 female) and Dataset II from 14 healthy participants (age: 32 ± 19 years; 7 male/6 female/1 not reported) with no neurological or psychological disorders.

### Experimental Paradigm

Participants were seated in a comfortable chair with room light on and were asked to look at a fixation cross on a black screen ~50 cm in front of them. A 5-min resting state data (Dataset I) or a 10-min resting state data (Dataset II) were recorded from each participant.

### Data Acquisition

fNIRS data were acquired using a multichannel continuous wave fNIRS system (CW6, TechEn Inc. MA, USA) operating at 690 and 830 nm wavelengths. The system is an optical imager with 32 frequency encoded lasers (half at 690 and half at 830 nm) and 32 avalanche photo-diode detectors. The light is carried from the system to the head probe and back via optical fiber bundles. fNIRS data were acquired at a sample rate of 50 Hz.

#### DATASET I: Optode Array and Auxiliary Measurements

##### Optode array

Both head optode arrays were designed utilizing AtlasViewer software (Aasted et al., 2015) (Figure 1). The optode array for Dataset I consisted of an elastic cap (EasyCap, Herrsching, Germany) with 8 sources, 12 long-separation detectors (~3 cm apart from the source) and 2 short-separation detectors (~1 cm apart from the source) providing, in total, 26 long-separation and 2 short-separation channels covering the occipital lobe.

##### Auxiliary measurements

Systemic physiological changes and head motions of the participants were simultaneously recorded along with the fNIRS data using an MP160 data acquisition and analysis system (BIOPAC Systems Inc., Goleta, CA). The pulse waveform was recorded using a PPG100C amplifier and TSD200 PPG pulse transducer placed on the participant's right index finger (BIOPAC Systems Inc., Goleta, CA). Respiration data was collected via measuring the abdominal (or thoracic) expansion and contraction using a RSP100C amplifier and a TSD201 respiration transducer (respiration belt) (BIOPAC Systems Inc., Goleta, CA) around the participant's chest. The blood pressure waveform was recorded using a DA100C amplifier and a TSD110 pressure transducer (BIOPAC Systems Inc., Goleta, CA) placed on the participant's right thumb. Head motions in x, y, z directions were collected using an accelerometer (ADXL335, Analog Devices Inc., Norwood, MA) secured on the head with a headband.

#### DATASET II: Optode Array and Auxiliary Measurements

##### Optode array

The optode array for Dataset II consisted of an elastic cap (EasyCap, Herrsching, Germany) with 16 sources, 24 long-separation detectors (~3 cm apart from the source) and 8 short-separation detectors (~1 cm apart from the source) providing, in total, 48 long-separation and 8 short-separation channels covering the head from frontal to parietal regions bilaterally.

##### Auxiliary measurement

Head motions of the participants in x, y, z directions were simultaneously recorded along with the fNIRS data using a 3-axis accelerometer (ADXL335, Analog Devices Inc., Norwood, MA) secured on the head with a headband.

### Adding Synthetic HRF to the fNIRS Data

**Figure 2:**
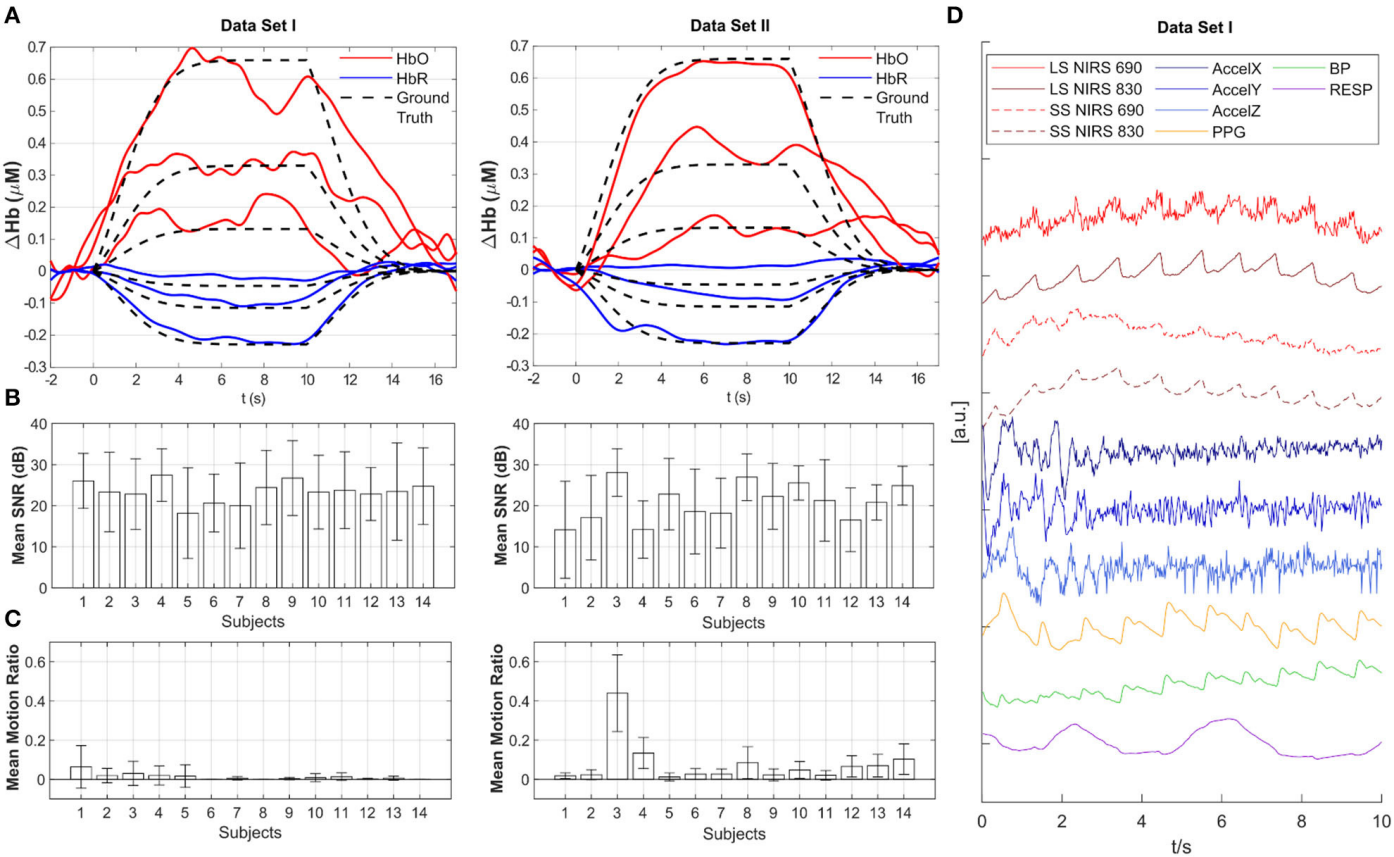
Overview of data quality and baseline analysis for Datasets I and II. **(A)** Across-trial recovered HRF estimated with tCCA GLM using Homer3 for all three amplitudes (100, 50, 20%) (participant 33 from Dataset I and participant 98 from Dataset II - the first participant in each data set). Red: HbO_2_, Blue: HbR, Black dashed: Ground Truth. **(B)** Mean SNR in dB across channels for all participants. Whiskers indicate standard deviation. **(C)** Mean Motion Ratio across channels for all participants which indicates the ratio of motion-contaminated data to the whole data. **(D)** Example time course of multimodal data in Dataset I. LS, Long-separation channel; SS, Short-separation channel; AccelX|Y|Z, Accelerometer; PPG, Photoplethysmogram; BP, Blood Pressure; RESP, Respiration.

In the documented data repository, we provide the acquired resting state data with and without synthetic HRF as well as the scripts used for the generation of the data to enable users to alter and re-generate ground truth HRF according to their needs. We generate synthetic HRFs with three different amplitudes using a gamma function with a time-to-peak of 6 s and a total duration of 16.5 s. The shape of this synthetic HRF is also depicted in Figure 2A. The three amplitudes are provided as percentages (100/50/20%) of a typical average amplitude of a task-evoked HRF (Huppert et al., 2006) and simulate varying degrees of CNR in the data: The (100%) amplitude is equal to +1% | −2% change from baseline intensity at 690 nm | 830 nm leading to an HRF peak amplitude of +0.66 | −0.23 μM for HbO_**2**_ | HbR, respectively with a differential pathlength factor of 6 (Delpy et al., 1988; Boas et al., 2004) for a 30 mm source-detector separation. For each participant in the two datasets, all resting state data is divided into windows of 20 s length. The HRFs are added in the intensity domain at a random onset (0–3.5 s) within each 20 s window for a randomly selected half of all available long separation channels after pruning with a 5 dB SNR threshold. This results in an average of 15 | 38 trials per participant and HRF amplitude in each resting state Dataset I | II.

### Data Structure and Format

Both datasets are presented in the Shared Near Infrared File Format V1.0 Specification (snirf), which is based on the HDF5 file format (https://github.com/fNIRS/snirf). ***SnirfClass***function loads the snirf object into the MATLAB environment. Table 1 provides the list of variables in the current dataset snirf object. The main fields of interest are: the ***data***field which has the fNIRS raw signal at each channel and relevant information, the ***probe***field which has optode array information, the ***aux***field which has all the auxiliary measurements and their details, and the ***stim***field which has the experimental paradigm information. Please note that, while snirf.aux(2)/(3)/(4) have AccelX, AccelY, and AccelZ measurements for both datasets, Dataset I has PPG, blood pressure (BP), RESP at snirf.aux(5), (6), and (7), respectively in addition to these.

**Table 1:** Snirf object fields.

**snirf.filename**	**Filename**
**snirf.fileformat**	“hdf5”
**snirf.data.dataTimeSeries**	Time-varying signals from all channels following the order in **snirf.data.measurementList** e.g., **10th** column of **snirf.data.dataTimeSeries** corresponds to **snirf.data.measurementList(10)** which is the channel defined with sourceIndex: 2; detectorIndex: 18 and wavelengthIndex: 1
**snirf.data.time**	Time
**snirf.data.measurementList**	Per-channel source-detector-wavelength information
**snirf.data.measurementList.dataTypeLabel**	Defined as (1) for HRF added channels and (0) for no HRF channels, specifically for this dataset e.g., to check whether HRF is added on channel 10 check **snirf.data.measurementList(10).dataTypeLabel**
**snirf.stim.name**	Stimuli labels
**snirf.stim.data**	Data stream of the stimulus channel
**snirf.probe.wavelengths**	List of wavelengths (in nm)
**snirf.probe.sourcePos**	Source position
**snirf.probe.detectorPos**	Detector position
**snirf.probe.sourceLabels**	String arrays specifying source names
**snirf.probe.detectorLabels**	String arrays specifying detector names
**snirf.aux.name**	Name of the auxiliary channel e.g., to check the content of an auxiliary channel 2 **snirf.aux(2).name**
**snirf.aux.dataTimeSeries**	Data acquired from the auxiliary channel
**snirf.aux.time**	Time for auxiliary data

## Baseline Analysis and Data Quality Assessment

Baseline analysis and data quality assessment was performed using the openly available Homer3 toolbox (https://github.com/BUNPC/Homer3) (Huppert et al., 2009). HRFs were recovered from the augmented resting state data using the processing stream provided in the repository (***tCCA_xmpl_procStream_Gauss_noHPF.cfg****under* **“*code”****folder*). This processing stream includes 0.5 Hz zero phase low pass filter with an effective order of 6, conversion to HbO_**2**_ and HbR using the modified Beer-Lambert Law (Delpy et al., 1988; Boas et al., 2004), and subsequent HRF estimation with the temporally embedded General Linear Model (tCCA GLM) approach using short-distance channels and a polynomial drift term for nuisance regression and Gaussian basis functions for the HRF regressor (von Lühmann et al., 2020a). Figure 2A exemplifies the resulting HRF estimates in one augmented channel for all three amplitudes from participant 33 from Dataset I and participant 98 from Dataset II. Data quality is provided for each participant as across-channel average of the Signal to Noise Ratio (SNR) in Figure 2B and as the mean motion ratio across channels in Figure 2C. Channel SNR is calculated as 20 × log10 of the mean over std. of the raw intensity signal. The motion ratio is calculated as the ratio between the cumulative time of segments in the data that were considered to be confounded by motion artifacts, as identified by the Homer2 function ***hmrMotionArtifactByChannel***(with tMotion = 0.5, tMask = 0.5, STDEVthresh = 20, AMPthresh = 5), to the total acquisition time. Figure 2D displays a typical segment of all available signals (z-scored) in the first participant in Dataset I. Long and short-separation fNIRS channels exhibit typical low frequency components and cardiac pulsation, which is also present in the PPG and BP measurement.

## Summary

We reported a multimodal fNIRS resting state dataset from 28 participants, that we provide with and without added synthetic HRF ground truth at three different amplitudes. We include the script used for the generation of these data to enable users to adapt this approach to their own needs. The availability of multiple auxiliary biosignals, such as motion (accelerometer) and PPG in the data, can be used to explore and extend existing multimodal fNIRS-based signal processing approaches (von Lühmann et al., 2019, 2020a). Resting fNIRS data with added known HRF enables the validation of novel processing methods for single trial HRF detection and BCI as well as more general artifact rejection and preprocessing approaches and their comparison with existing methods. This can also be useful for methods that tackle challenges such as non-stationarities in the amplitude and time to peak of hemodynamic responses to a stimulus.

## Data Availability Statement

Publicly available datasets were analyzed in this study. This data and the code for HRF ground-truth generation can be found here: https://www.nitrc.org/frs/?group_id=1071.

## Ethics Statement

The studies involving human participants were reviewed and approved by Boston University IRB. The participants provided their written informed consent to participate in this study.

## Author Contributions

XL and NG collected the first and second datasets, respectively. AL and MY analyzed the data, prepared the figures, and wrote the report. DB reviewed the report. All authors confirmed the final version of the report.

## Conflict of Interest

The authors declare that the research was conducted in the absence of any commercial or financial relationships that could be construed as a potential conflict of interest. The authors declare that this study received funding from Facebook. The funder was involved in the decision to submit the paper for publication.
